# Difficult conversations: Australian Indigenous patients’ views on kidney transplantation

**DOI:** 10.1186/s12882-017-0726-z

**Published:** 2017-10-11

**Authors:** Jeannie Devitt, Kate Anderson, Joan Cunningham, Cilla Preece, Paul Snelling, Alan Cass

**Affiliations:** 1 Menzies School of Health Research, Darwin, Australia; 2 Cairns, Australia; 3 Royal Prince Alfred Hospital, Sydney, Australia

**Keywords:** Kidney transplantation, Indigenous Australians, Health inequity, End stage kidney disease

## Abstract

**Background:**

Indigenous Australians suffer a disproportionate burden of end stage kidney disease (ESKD) but are significantly less likely to receive a transplant. This study explores Indigenous ESKD patients’ views on transplantation as a treatment option.

**Methods:**

The Improving Access to Kidney Transplants (IMPAKT) research program investigated barriers to kidney transplantation for Indigenous Australians. An interview study, conducted in 2005–2006, elicited illness experience narratives from 146 Indigenous patients, including views on transplant. Interviews were conducted at 26 sites that collectively treat the majority of Indigenous ESKD patients. Key themes were identified via team consensus meetings, providing a flexible framework and focus for continued coding.

**Results:**

Four inter-related themes were identified in patient commentary: a very high level (90% of respondents) of positive interest in transplantation; patients experienced a range of communication difficulties and felt uninformed about transplant; family involvement in decision-making was constrained by inadequate information; and patients needed to negotiate cultural and social sensitivities around transplantation.

**Conclusions:**

Indigenous ESKD patients demonstrated an intense interest in transplantation preferring deceased over living kidney donation. Patients believe transplant is the path most likely to support the re-establishment of their ‘normal’ family life. Patients described themselves as poorly informed; most had only a rudimentary knowledge of the notion of transplant but no understanding of eligibility criteria, the transplant procedure and associated risks. Patients experienced multiple communication barriers that - taken together - undermine their engagement in treatment decision-making. Families and communities are disempowered because they also lack information to reach a shared understanding of transplantation. Cultural sensitivities associated with transplantation were described but these did not appear to constrain patients in making choices about their own health.

Transplant units and local treatment providers should collaborate to develop user-friendly, culturally informed and region-specific patient education programs. Quality improvement cycles should underpin the development of national guidelines for patient education.

Noting Indigenous patients’ intense interest in transplantation, and nephrologists’ concerns regarding poor transplant outcomes, research should prioritise exploring the predictors of transplant outcomes for Indigenous Australians.

**Electronic supplementary material:**

The online version of this article (10.1186/s12882-017-0726-z) contains supplementary material, which is available to authorized users.

## Background

Indigenous Australians suffer a disproportionate burden of end stage kidney disease (ESKD) at an earlier age than non-Indigenous Australians [1]. The recognised optimal treatment for most patients is a kidney transplant, however Indigenous patients are both significantly less likely than non-Indigenous patients to receive a transplant and slower to be wait listed [2]. Although a focus of research attention for over a decade [3], the disparity in access persists [4–6], playing out in a context of chronic national organ scarcity [7]. Internationally, comparison with Indigenous groups in similarly high-income countries including Australia, Canada, New Zealand and the United States has shown that irrespective of health care systems differences, all Indigenous patients are both less likely to receive a transplant and more likely to wait significantly longer for a transplant than “white” patients [2].

Patients deemed suitable by their treating specialist and relevant transplant unit are listed on the national transplant waiting list. Alternatively a living person, usually a family member, can donate a kidney – referred to as living kidney donation (LKD). However, with multiple co-morbidities, as well as high rates of chronic kidney disease, LKD presents significant impediments for Indigenous people [8, 9].

Indigenous patients face multiple challenges to accessing transplantation [2] via a pathway recently described as *fragmented, confusing, isolating and burdensome* [10]. High rates of late referral lead to poor physical condition at commencement of ESKD treatment [11] and complications in ensuing phases of treatment, including consideration of transplant suitability [12]. There is also an absence of user-friendly, culturally-informed processes and strategies to educate and support Indigenous patients, families and their clinicians in essential, participatory, decision-making about transplant and organ donation [13]. Along with a commonly held perception that Indigenous ESKD patients are generally less ‘compliant’ with treatment requirements [5] nephrologists also have on-going concerns about Indigenous transplant outcomes [14, 15].

Locality, in the sense of state or jurisdiction, has been associated with variability in access to the waiting-list as well as transplant in both Australia [16] and the United States [17]. In Australia, locality, in the sense of distance – referring to the proximity of communities to dialysis and transplant treatment facilities and/or remoteness – referring to the relative range and density of locally available services – impacts negatively on the treatment trajectory of most Indigenous patients. Despite the acknowledged effects of locality on accessing dialysis services [18, 19], and the association of rural location with poorer transplant outcomes [20], distance and remoteness have not been systematically examined as potential influences on Indigenous patients accessing transplant.

All Australian transplant units are located in metropolitan hospitals in southern and south eastern Australia, whereas around half of all Australian Indigenous people with ESKD undertake dialysis many hundreds of kilometres away in central and northern Australia [21]. Treatment in a facility with a transplant unit has been shown to significantly increase likelihood of transplantation. Reasons for this may include the characteristics, size and organisational aspects of the centre, easier access and, highly-trained health care staff with a more positive attitude towards transplantation [22].

With the exception of a minority doing home-based dialysis [11], patients from Australia’s remote areas have extremely limited access to dialysis services in their homelands. The majority therefore need to relocate permanently to distant regional dialysis centres. The profound social, emotional and cultural dislocation this causes has been extensively reported for over 20 years in Australia [23–29] and – because of some geographic similarities – also in Canada [30].

The experience of dislocation is exacerbated by the ‘*vast cultural and linguistic distance’* between dialysis patients and families on the one hand and their predominantly non-Indigenous health care providers on the other [27, 28]. The linguistic and communicative context is complex with over 100 extant Indigenous languages [31, 32]. Communication and related issues dominate dialysis patients’ reported commentary including difficulties in acquiring information about their illness and treatment options, exclusion from information, insufficient time/opportunity for full discussion and over-reliance on medicalised terminology [33].

Providing, or not providing, specific transplant information for patients is a contributing factor in disparities in access to transplant [34]. Improved, standardised patient education is associated with increased access to transplant [35]. Interestingly, in a UK study where linguistic/cultural difference and minority group status were not directly implicated, patients reported being uninformed of their transplant situation and dissatisfied with prevailing communication practices [36]. Concerns in common with Australian Indigenous patients included insufficient time for discussion, too much/poorly timed information, difficult medical terminology, insufficient opportunities for revisiting treatment decisions, and being excluded from treatment decisions and discussions. Cultural and linguistic differences, however, are specific to particular populations. Recent research demonstrated that culturally and linguistically tailored transplant education programs [37] employing multiple strategies/activities [38] lead to more informed patients and increase access to the transplant waiting list. However, and perhaps more importantly, while well-identified, specific barriers may not be unique to Australian Indigenous patients, “*few other populations experience the combined, interactive and continuing effects of all these factors”* [original emphasis] [25].

### Patient preferences as a barrier to transplant

International literature has long reported on diverse communities’ knowledge and attitudes towards kidney transplant and organ donation [39–41]. However, recent studies suggest that patient aversion or disinclination to transplantation - whether for cultural, social, religious, health or other reasons – may also contribute to differential access by particular groups [36, 42, 43]. African American dialysis patients, for example, reported reluctance to seek transplant due to perceived burden of medications, possible organ failure, satisfaction with dialysis [44] or concerns about more general risks and the equity of the organ allocation process [45, 46]. Data from the United Kingdom suggests that particular patient groups are also reluctant to seek or accept an LKD transplant [47]; other research reports patients speaking of feelings of ‘guilt’ or concerns over the LKD donor’s subsequent health [36].

Excepting two small, early studies [26, 27] little is known of Indigenous Australians’ views on kidney transplantation or organ donation – either from community or patient perspectives. A potentially comparable Canadian study [30] reports only indirectly on Indigenous patients’ views through an analysis of commentary provided by clinicians working with Indigenous ESKD patients. Our study begins to address that gap, exploring – through in-depth interviews – Indigenous Australian ESKD patients’ knowledge of, and attitudes towards kidney transplantation, as well as their experiences of progressing/not progressing towards it. The research was conducted in 2005–2006 as a sub-study of the broader IMPAKT (Improving Access to Kidney Transplants) research program investigating barriers to kidney transplantation for Indigenous Australians [3].

## Methods

The IMPAKT research program was collaboratively designed and implemented by an experienced, multi-disciplinary research team, including nephrologists, an Indigenous health practitioner, epidemiologist, anthropologist and a post-graduate research student. A model of essential steps on the pathway to transplant [48] provided a conceptual framework for the research program (see detailed description of protocol [32]. The extended narrative-style interview with its potential flexibility is a qualitative method well suited to exploring individual lived experience [49]. This paper reports on Indigenous ESKD patients’ views on transplant and organ donation.

### Ethics

The study was approved by 14 jurisdictional ethics committees, including five all-Indigenous committees (see Additional file 1). The National Aboriginal Community Controlled Health Organisations (NACCHO) supported the project, provided advice on involving Indigenous ethics committees and facilitated engagement with local Aboriginal Health services. Site-based reference groups, including staff from local Aboriginal community-controlled health organisations represented staff and institutional interests.

### Research settings

**Fig. 1:**
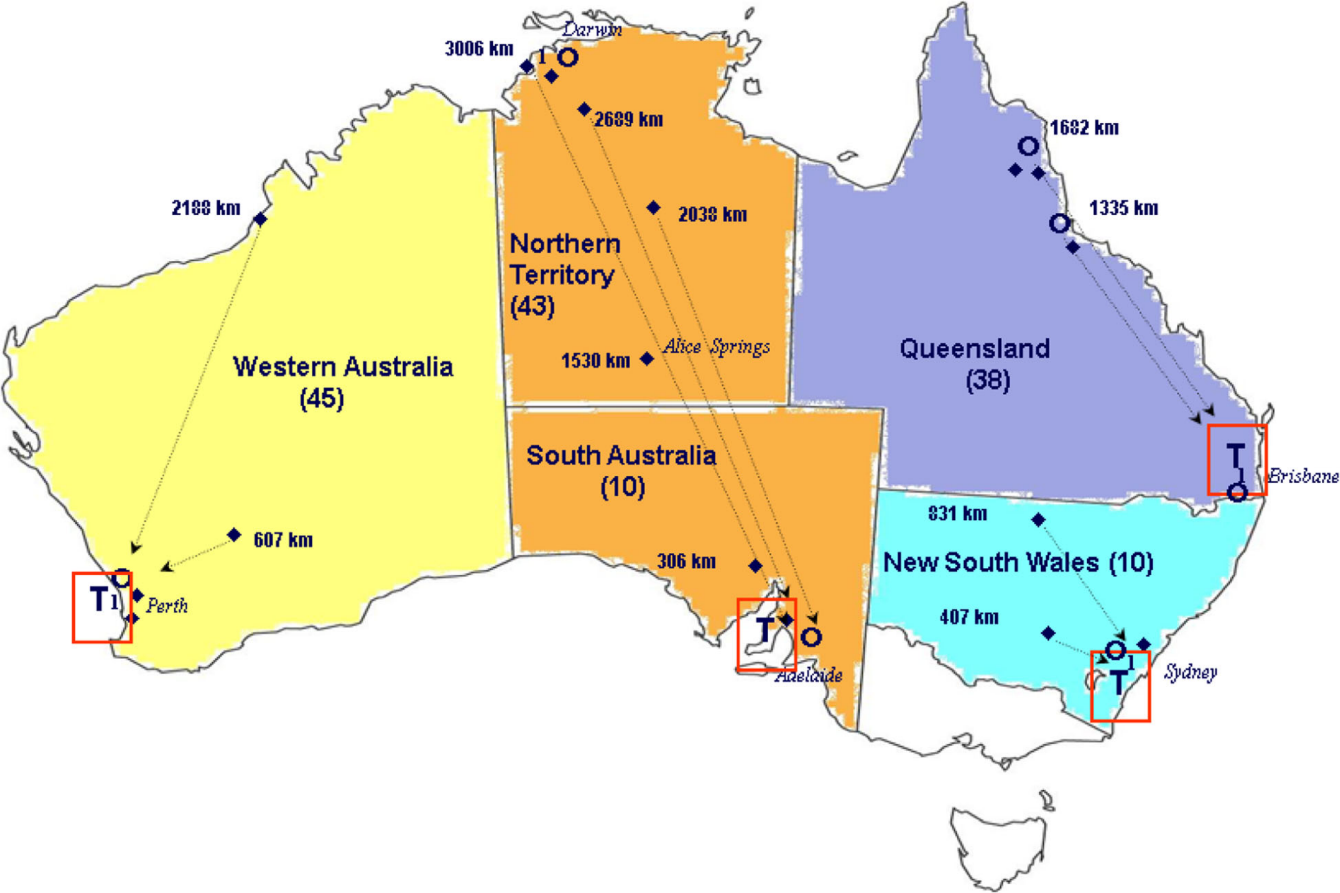
Relative locations of participating transplant networks and number of Indigenous interviewees per state. T = Transplant Hospital site. Ο = hospital dialysis site. ♦ = satellite dialysis site. (45) = number of interviewees in state/territory (source: authors)

The IMPAKT project established partnerships with the networks of Australian hospital transplant units and dialysis treatment centres that collectively treat the majority of Indigenous ESKD patients. The 26 research sites constitute four separate transplant ‘networks’, each with its metropolitan hospital transplant unit and associated regional (hospital and satellite) dialysis treatment centres (Fig. 1). Participation in the study was voluntary; interviews were conducted at each of these 26 sites.

### Recruitment

A maximum diversity sampling strategy guided patient recruitment. Applied site by site, this sampling strategy aimed to include:

- full range of treatment modalities (including transplant);
- patients with dialysis start date of less than 5 years prior;
- age between 18 and 65 years;
- balance of genders; and
- Indigenous and non-Indigenous patients (the latter not included in this report)

The recruitment process involved both staff and interviewers (Table 1) and all patients recruited participated. No figures were kept on numbers of patients declining to participate and participants were not compensated.

**Table 1:** Sequence of recruitment and informed consent process

Stage	When/Where	Activity
1	3 weeks pre-field work	Project staff send recruitment guidelines to participating site; staff begin identifying potential participants
2	On-site	Staff enquire if patient is interested in hearing about study
3	On-site	Staff introduce IMPAKT interviewer to interested person, or provides patient contact details
4	On-site	Interviewer explains project to patient, provides patient information sheet
5	On-site	Interviewer re-visits patient; if willing to participate, they nominate interview time
6	On-site	Interviewer meets with patient, completes informed consent paperwork, conducts interview
7	Home base	Interviewer sends transcript of interview to participant, including letter of thanks; participant given 6 weeks to amend transcript

### Data collection and analysis

Interviews were conducted individually and face-to-face (with one exception by phone) by three investigators (JD, CP, KA). They were digitally recorded and transcribed. Using a narrative format, interviewers elicited accounts of patients’ illness experiences, including effects on family life, views on transplant, views on being informed, and their satisfaction with health services. Patients were invited to provide their views irrespective of their personal health and/or likelihood of a transplant. Importantly, interviewers also made clear that participation had no influence on transplant potential.

Patient interviews were based on a thematic core which underpinned the overall IMPAKT project. Themes relevant to the patient interviews included: information and communication processes; decision-making and treatment options; attitudes and views on transplantation. However the patient interview itself was structured as a life story narrative; patients were invited to describe the sequence of what had happened to them, how it had affected them, and their understanding of their current options. Interviewers worked with a topic list which included the following: personal health history; social and psychosocial context; attitudes, values, treatments, information and communication, transplant and, satisfaction with services. Interviews were 30–40 min long on average [32].

Indigenous patient interviews were conducted predominantly in English. A subset of those interviews (seven) was conducted entirely in Pitjantjatjarra (a Central Australian Indigenous language) as a comparison.

The research team met regularly throughout the study and major thematic categories were proposed, discussed and agreed on via consensus. The analytic methods used were evolutionary and iterative in nature [50]. Two investigator/interviewers (JD, KA) coded all interviews. Participant demographics were self-reported. Interview data analysis was supported by QSR NVivo 7 and NVivo 11 (Doncaster, Australia). Descriptive statistics were generated using SPSS 15.0 for Windows (Chicago, Illinois).

## Results

### Participants’ profile

A total of 146 Indigenous ESKD patients were interviewed. As a group they were relatively young with almost half (46.6%) less than 50 years of age and women slightly outnumbering men (Table 2). Many patients (52.1%) were also supporting dependents. Time since starting dialysis ranged from less than 1 year to more than 10 years with the majority (73.3%) having been on dialysis for 5 years or less. Although 93 participants (63.7%) reported that they were employed prior to starting dialysis, only 14 (9.6%) were employed at the time of interview; a majority (70.5%) were living in rented accommodation. Of the total group, 52 (35.6%) had either no formal schooling or primary level only while 20 patients (13.7%) had completed either secondary school or some post-secondary education. Over half of participants (*n* = 84, 57.5%) reported speaking an Indigenous language as their first language; this included a range of distinct Aboriginal and Torres Strait Islander languages as well as Aboriginal English and Creoles. Of those who did not speak English as their first language (*n* = 84), almost all (*n* = 81; 95.3%) reported English as their second language.

**Table 2:** Demographic and socio-economic characteristics of Indigenous patient participants (*n* = 146)

	Number	Percent
Age (years)		
20–29	5	3.4
30–39	19	13.0
40–49	44	30.1
50–59	50	34.2
60–69	21	14.4
≥ 70	7	4.8
Gender		
Female	76	52.1
Male	70	47.9
Place of interview		
New South Wales (4 sites)	10	6.8
Northern Territory (5 sites)	43	29.5
Queensland (6 sites)	38	26.0
South Australia (3 sites)	10	6.8
Western Australia (5 sites)	45	30.8
Has dependants^a^		
Yes	76	52.1
No	68	46.6
Current accommodation^a^		
Own home	17	11.6
Rental accommodation	103	70.5
Other^b^	25	17.1
Highest level of education^a^		
Post-secondary	13	8.9
Completed secondary school	7	4.8
Some secondary	71	48.6
Primary only	35	24.0
No formal education	17	11.6
Time since dialysis start^c^		
< 1 year	28	19.2
1–2 years	29	19.9
3–5 years	50	34.2
6–10 years	37	25.3
> 10 years	2	1.4

^a^Number missing: has dependants (2); current accommodation (1); highest level of education (3)

^b^Includes hostel, nursing home and staying with family

^c^includes patients (4) with functioning graft at time of interview

Participants were predominantly, but not exclusively, from regional and remote Australia. The place of ‘usual residence’ for over half of the patients was sufficiently distant from a treatment centre that permanent relocation to access dialysis was required. The place of usual residence for almost three quarters (74%) of the patients was more than 1000 km from the relevant network transplant hospital (Table 3). The majority (91%) were undertaking in-centre haemodialysis.

**Table 3:** Distance from patients’ usual residence to (a) dialysis treatment facilities or, (b) transplant hospital

Distance^a^ (kms)	(a) Usual residence to dialysis facility		(b) Usual residence to transplant facility	
	Patients (*n*)	%	Patients (*n*)	%
0–49	51	36	11	8
50–299	34	24	4	3
300–599	23	16	17	12
600–999	23	16	5	3
1000–1999	9	6	67	47
> 2000	2	1	38	27
Unassigned	4		4	
Total patients	146		146	

^a^Distances between patient ‘usual residence’, treating centres and transplant units respectively was calculated using Google Maps (http://maps.google.com.au ‘Get Directions’ function) and an Australian Government web tool at Geoscience Australia www.ga.gov.au

Four major inter-related themes were drawn from patient commentary:

- interest in transplant as a treatment option;
- becoming informed and communicating with clinicians and carers;
- family support in transplant decision-making; and
- negotiating cultural sensitivities.

#### *“I really want one”*: Interest in transplant as a treatment option

Despite the dearth of appropriate information and communication difficulties, 115 (90%) of 132 Indigenous patients who gave a view, expressed a definite interest in a transplant. The most frequently cited reasons patients gave for being interested in transplant was associated with the potential of a transplant to restore their life to ‘normal’ and, in particular, to ‘get home’ and be with their families:

*I feel that a transplant will allow me to get back to a normal life.* (2-041)

*If I could get one, maybe, I’m happy to go back home.* (3-009)

*I really want one. I really want to go home.* (3-174)

Most understood transplant as the only escape from a lonely life regulated by dialysis sessions:

*I’d love to get a transplant and get off this machine.* (3-155)

*I want to get out from here and go back home.* (2-026)

The group of patients that were interviewed in their first Indigenous language, expressed an equally strong interest in transplant:

*I can’t go [home] and I’m really missing my friends and my family… I’m getting used to living in town now - but in my spirit I really want to be able to go home. …The most important thing to me at the moment is that I do the right thing and [then] be able to get a kidney… because I’m really suffering.* (3-178)

Nine other patients were not currently interested, but were not opposed in principle. Reasons for their lack of interest included believing themselves to be too old (3–106, 3–025, 2–020), health uncertainties (2–115), uncertainty about what transplant entailed (2–061), preferring to manage dialysis (2–016, 2–005) being undecided (2–027) and being frightened (2–088).

Four patients reported either no interest or active opposition to the idea of transplant. Their reasons included: *not believing in it* (2–030), that transplant *was going against my (Christian) faith* (2–009), being fearful of harm from the deceased donor’s spirit (4–040) and preferring to *have my own kidney and live the way I am* (3–130). (Four current transplant recipients were among those who discussed transplant but were not included in these totals).

##### Living Kidney Donation (LKD)

Many patients were aware of the concept of LKD but frequently pointed to the poor health of family members, mentioning particularly diabetes and heart problems. Some reported refusing offers of kidneys from relatives – particularly adult children. A common reason for refusal was the likelihood of the donor succumbing to kidney disease in the future. In addition, patients cast potential recipients, including themselves, in the role of a ‘*taker*’ – even a ‘*spoiler’* of another person’s life: *I don’t want to spoil my family* (2–044); *I couldn’t do it to him [brother]* (2–105). A man who thought the notion of transplant ‘a good idea’, but had reservations about LKD explained more fully:

*Well it’s a new thing ...these kidney transplant things, you know, getting other people in there, taking things. He [the donor] gets scared himself for giving it [the kidney] away you know - taking some life from another person - you don’t know what’s going to happen to them. ... They have to live a normal life too you know.* (4-022)

Another woman recounted refusing her son’s offer of a kidney despite his assurances that it was possible to live on one kidney:

*I said, “Yeah, but say if it fails, then you’ve got one kidney left and it might be no good.” I mean, who knows...In years down the [track], their kidneys might fail and [but for] that one kidney that you might take off them, they could be alive. So this is what I’m looking at, you know.* (4-005)

A respondent (speaking in his own language) suggested that even making such a **direct** request to family members entailed an unconscionable degree of emotional ‘force’.

*I: No, I haven’t talked to them about it. I’m leaving it up to them. If they want to give it, they can do it, I don’t want to force them.*

Q: Is that an Anangu [Aboriginal] way - that you don’t go asking someone because that would imply that you’re forcing them to make a decision?

*I: Well, yes, I just don’t go around asking, I think that’s their decision to make, and their body, and they’ve got to decide. So I haven’t talked to my family about it - not yet. It may be that they get sorry for me, that they’re sympathetic to my position and that’s it.* (translated from Pitjantjatjarra) (3-178)

There was also some concern expressed that receiving a live donor kidney could entail obligation into the future.

*But … some families, if [they] give you something, they want something you know…I woke up to that.* (2-053)

*I wouldn’t mind if it [LKD offer] was genuine - from the heart you know … that’d be fine. But I feel with the younger brother, maybe he’d be on my back, you know like, “I don’t give you this [for nothing]” - and the other [brother], well he’d definitely want something - he’s that sort of bloke you know, I’d have to give him me TV or me bloody whatever.* (2-105).

#### *“I have no idea”:* Becoming informed and communicating with clinicians and carers

Of 127 Indigenous patients explicitly asked, 59 (almost half) described themselves as not being well informed and/or not understanding important aspects of their treatment or not understanding their care providers, particularly kidney specialists. While some understood the notion of ‘transplant’ in broad outline, the procedure, implications and the process of actually getting a transplant was unclear for the majority. Moreover, they were uncertain about the eligibility of Indigenous people for transplant. Crucially, patients did not know when, how or with whom they should/could properly raise the subject.

*When I first came in with kidney failure … I didn’t really get much information at all. It could have been much better than it was… Now it’s two years later and I’m just starting to find out about transplant... I don’t know anything about it, or how people get on the list…One day I noticed that they (three fellow Aboriginal patients) were missing from the unit …I found out that they got transplants…that’s what got me asking some questions. Now my family are talking about a transplant… We need to talk together about this and we all need information about what donating a kidney involves.* (3-111)

This situation was reported by an urban Aboriginal man dialysing at a large metropolitan centre associated with a transplant hospital. This patient, even with advantages including English as a first language and proximity to a transplant unit, recounted having to navigate the health system by personal detective work.

An indicator of patients’ difficulty was their lack of understanding of their status in relation to the transplant waiting list. Of 137 Indigenous patients responding to a question asking whether they were currently on the transplant waiting list, approximately one in four were either mistaken or uncertain about their waiting list status.

Asked if she was on the transplant list, one patient commented:

*I have no idea either - I don’t even know that! Well, to tell you the truth, I don’t even know what transplant they’re talking about - they just say ‘transplant’, you know, ‘kidney’.* (4-032)

Clinicians may well have spoken to some patients, but little had actually been communicated. Patients spoke of various communication problems with kidney specialists, including the complexity of the content, of specialists speaking too fast, of being overly assertive and of a perceived reluctance of specialists to spend time speaking with them:

*They [staff] don’t give it [information] the right way. Instead of like trying to teach them, they come across like they know everything and they don’t compromise on that, hey? When they come across like that everyone’s too scared to ask them questions why, so then they just shut up and think, “Well I’ve been told this, so that must be it”.* (4-015)

*Well they don’t like to tell us - like the doctors even themselves don’t want to say anything but I think it is our right to know; he [doctor] said, “It [kidney] is just not working… it’s not that good, you know”. That’s all they said…Well they sort of avoided most of the questions, until I got really cranky with them [then] they started telling me what’s going on and that. But they got a very funny way of communicating with people*. (3-021)

Confused by an apparent lack of any further action or discussion, patients might easily conclude that they must have misunderstood in the first place:

*The doctors tell me about kidney transplant but I don’t understand what he says – he tells me I’m on the list but nothing is happening.* (2-075)

Even a patient with a willing, potential living kidney donor was perplexed:

*But we (family), that’s all, we talk, we don’t know where to go or what! Where to ask or what!* (2-029)

Most patients reported not questioning staff or seeking clarifications. Even patients actively seeking information felt intimidated in such a large, unfamiliar and busy institutional setting:

*I was asking [nurse] now at the hospital…I’d really like – you know - see if I can get a transplant…. I just asked, you know. She didn’t say she’d give me any information.… she told me she’d talk to the doctor, but I don’t - you know, we haven’t got - we have to get - what do you say - to see a doctor?* (3-026)

Asked what he knew about his situation, one respondent drew a key distinction between ‘being informed’ about regular dialysis sessions and having a plan for the future:

*Well we don’t know. We really only just go in and out and have the treatments…So we don’t know whether we’re getting a bit better or things are getting a bit worse. They don’t tell us whether we’re improving or getting worse. We’re just going in and out of the [dialysis] sessions.* (translated from Pitjantjatjarra) (3-178)

Reflecting on the unsatisfactory way information and/or education is provided led some patients to suspect that clinical staff used their greater power to restrict patient access to information:

Q: Why haven’t you gone and asked them at the renal unit or somewhere, to clarify the story…gone and knocked on the door and asked?

*I: You don’t go knocking on their door, [it’s a] ‘danger one’*

Q: What do you mean ‘danger’?

*I: The door is locked. They sit behind closed doors.* (translated from Pitjantjatjarra) (3-179)

This man went on to explain further:

*I – we would like to be spoken to clearly in an understandable way by doctors – …by doctors who like Anangu (Aboriginal people), by understanding [empathetic] doctors who talk - they’re good – a lot of other doctors can’t talk with us… their talk is hard [to understand].*

He understands the nurses, they *speak nicely,* and he has let them know he is interested in a transplant. They advised him *to go and ask the specialists.* He also noted that he had never received anything on paper and that this interview was the first occasion in his 3 years of treatment where he had access to an interpreter.

Indigenous patients are from culturally diverse geographic and language groups; they value both their own and other patients’ privacy. Many noted that they usually do not discuss their health situation with other patients. In part, this reflects an understanding that senior clinicians, rather than other patients, have the relevant knowledge.

*Patients - we don’t talk to each other about that kind of thing (transplant) – mainly we talk to the doctors. I want him to tell me everything.* (3-093)

*I don’t talk to the other patients about dialysis cause they are in the same boat as me.* (2-009)

But there is also sensitivity to the possibility of unwittingly giving offence to other patients or their families, as happened to this man:

*[Patients] don’t talk to each other…I asked one of the fellas, I said, “I’m looking at having a kidney transplant but I’ve got to lose weight [first].” “Anyway” … I said, “what about you - you looking at getting a transplant?” He said, “No”. He turned around and looked at me straight in the eye and said, “No … One of my family had passed away getting a kidney transplant”. Then I shut up.* (3-053)

Despite patients regularly interacting with knowledgeable staff, multiple barriers confound effective communication. Nurses are a potential source for preliminary transplant information; however, together with their other professional priorities, nurses may have reservations about discussing transplant with Indigenous patients, including not wanting to raise expectations, or (unknowingly) offend cultural protocols. When asked if patients queried her about transplant, an Indigenous nurse in a regional centre with an all-Indigenous patient group replied:

*No, transplant’s never brought up, never spoken about.... I don’t know, maybe because there’s not enough information about it to our patients, it’s not something we talk about really; we’re busy doing everything else. Transplant is last on the list!* (3-127)

Asked whether she thought the treatment centre promoted transplant to potentially suitable Indigenous patients, a transplant-hospital-based Indigenous liaison officer hesitantly explained:

*I don’t think they [renal nursing staff] do and I think we can … I think it’s a cultural thing too. They [renal staff] don’t know whether it’s culturally appropriate to even ask that sort of thing.* (2-035)

#### *“We all need information”:* Family in decision-making

Patients described themselves as poorly informed about transplant as a treatment option. Their families and home community members have far fewer opportunities to learn about the transplant treatment, yet they provide the primary support network for patients. The dialysis patient (cited earlier) who only ‘discovered’ the transplant option by noticing fellow patients apparently ‘disappearing’, articulated the situation clearly:

*Now my family are talking about a transplant. They need some information. We need to talk together about this and we all need information about what donating a kidney involves. It is a bit hard to talk about it though because my family doesn’t get together that often.* (3-111)

Through their illness and treatment experience, patients may come to a view on transplant that is not necessarily shared by their distant families. Although reporting themselves as being interested in transplant, patients also reported family and community reservations about transplant. Some described extended, stressful negotiations to achieve family consensus around the transplant treatment option. There is fear about the operation itself and the possible death of the family member in a faraway city.

*And I’ve talked to people from outside, like, whose got good kidneys, and they said, “Don’t go on it, don’t put yourself on the list”. And they kept talking me the other way. “It’s a one-way thing … if you have an operation, you’re finished, that’s it”. They refusing me [to do it]. They don’t like the idea, especially the family... I’m keen to have it. I’m excited to have it.* (3-086)

Some drew a distinction between their own close kin/family and a more generalised Aboriginal community view (from *people outside [who have] good kidneys*) reported to, or known by, patients.

*They (community members) saying all this rubbish about, “You’re getting that kidney from that dead man!” You know, something like that and they start to make you more frightened… That’s what they’re trying to do to you. But don’t listen to them, it’s your decision, it’s your mind, make your own mind up and do whatever you feel for you. .. I don’t listen to them other mob of people when they talk about it… they don’t know anything about that, you know…* (3-091)

Both of these speakers point out that family and/or other community members who are ***not*** on dialysis do not really understand the patient’s situation and options. On the other hand, relatives’ fears of possible negative outcomes, including death, serious cultural transgression or psychic fragmentation, reflect their care and deep concern. Patients described a situation where a lack of shared knowledge about the transplant process feeds into fear, for both themselves and their families.

Some patients also reported reducing family anxiety and fear by avoiding the transplant topic altogether.

*I don’t talk to them [family] about my condition you know. I think they’ll be scared eh?* (4-022)

However, not all were fearful:

*..as long as those kidneys are good and can be used then you’re okay. ... I’m completely confident…I’m not afraid. If I’m going to be afraid then I’m likely to be here forever in this state [of illness]… So because of that, I’m approaching it confidently (translated from Pitjantjatjarra)* (3-177).

The majority of Indigenous patients knew or had heard about other patients who had received transplants. They described a range of scenarios, including recipients who (apparently) were doing well, others who had died shortly after their transplant and yet others who had been unwell throughout the transplant period. In virtually every instance, the speaker asserted that nothing they had seen or heard – either good or disappointing outcomes – altered their own interest in a transplant. Seven of ten previous (failed) transplant recipients were interested in another transplant and only one of the ten had ruled it out absolutely.

#### *“It’s a new thing”:* Negotiating cultural sensitivities

Patients frequently alluded to the potential complexities and sensitivities that flow from the cultural, social, emotional and spiritual dimensions of this ‘new’ concept of transplantation.

*[Someone] should tell the doctor because some Aboriginal people who are dialysing, they are finding it very hard from our own family, because that’s what the ancestor say. They (family members) say, if they give us a kidney and we die middle of that, maybe after 9 years, or whatever, we pass away and we’ve got their kidney. They think we will haunt them… And no-one has explained that to the doctors.* (3-086)

This man explains his (and some other patients’) difficulties. His relatives are telling him that a person should keep their own body intact – because that’s *what the ancestors say*, (i.e. it’s a cultural tradition). His relatives advise him that bad outcomes will follow a transgression of their tradition, for example, the deceased patient’s spirit will haunt or otherwise disturb the donor.

However, he remains keen on the idea of transplant and, along with many other patients, sought to resolve the impasse by invoking another traditional rubric – that of a person’s right to autonomous action in relation to their own person, their own body and, by extension in a contemporary frame, their own health:

*Now I have to get a kidney from someone else…That’s my decision. It’s my body and I want to go ahead. It doesn’t matter if I live or die…It doesn’t matter what nationality we are, white, black, brown, whatever, we all come from the ground. In old way, we call the mother ‘earth’, dust to dust, so that’s where we come from, doesn’t matter.* (3-086)

*Yeah, if the doctors say my heart is right, I’ll say “yes” for my transplant. I’ll agree to whatever they want, for myself anyway, it’s not for anybody else, but for myself.* (3-164)

*I’m choosing my own choice, I’m choosing the transplant… I said, “Yes, that’s me, that’s my identity!” (laughs)* (3-098)

*Family got nothing to do with it. You decide, you know what to do. You want to go through the operation, well it’s up to you, nobody can tell you what to do… that’s your body and that’s your kidney -if you want a new one, you ask the doctor and the doctor will tell you. Don’t get fright - some people get frightened eh?* (3-160)

Asked whether any cultural issues constrained patient transplant choice, one patient suggested pragmatically:

*No, some may have [reservations], but there is* ***that*** *many people with renal disease that that should overcome any cultural things, you know…There probably would be a little bit of fear just because there’s not enough education on it, you know.* (3-019)

Patients who were Christians had a faith-based interpretation of their situation. This provided both personal and spiritual strength within a framework that promoted universal help, under-cutting notions of wrongdoing through accepting donated and/or non-Indigenous organs:

*We trust in the Lord, He’ll find a kidney for me; I’m a Christian person - a Baptist – and people always pray for us dialysis patients. I’m not frightened. I don’t worry if that kidney comes from a White person or someone else – it doesn’t matter…God made people to help each other. When the doctors get that same blood, same numbers, that kidney will be alright.* (3-166)

Even in these different contexts an emphasis on individual agency and autonomy supported patients to make decisions that others – including family or other patients – opposed.

*[B]ut for me I trust myself to go ahead with the transplant; I trust myself. I have faith you know, I believe in the Lord Jesus* (3-164)

The man who had inadvertently annoyed a fellow patient who was anti-transplant, also emphasised his own right to decide:

*No. That’s what I think and [when I] make a decision, that’s my business you know. I’m not going to have someone coming and trying to convince me not to go ahead with it [transplant].* (3-053)

The source of a patient’s transplanted kidney was a topic of interest and considerable speculation by fellow patients. However, the dearth of accurate, timely information and limited communication opportunities, left patients floundering; scraps of hearsay and misinformation circulated among co-resident patients:

*All I know is that when I was staying at the hostel here a lady came down from Alice Springs for a transplant and when she went back a couple of months ago, she passed away. Yeah that’s what I’m scared of, but her transplant was from - not from her family – [it was] from a white person - I don’t know.* (2-061)

*One of those transplant workers says you have to get it [kidney] from an Aboriginal person, they got the same donor’s blood group or something.* (3-026)

*Nope. Because as far as I’m concerned, we’re all the same inside anyway - we’ve all got the same parts. I’m just grateful that I can be on the list.* (3-072)

Asked whether it was acceptable for her as an Aboriginal woman to receive a kidney from a ‘white’ (non-Aboriginal) person, this woman was clear:

*It’s a good way to get one like that. All my family, my sister, all my younger siblings, they’re all diabetic, all sick. Maybe all of us are sick…Yes [so] a white man is OK, or a white woman. Aboriginal people are OK too, but they’re all sick. There are a lot of well white people so it is good to get one from them. (translated from Pitjantjatjarra)* ( 3-175).

Patients and families held a range of views as to the acceptability and suitability of donors of different ethnicity and/or gender to the patient. A few raised concerns that donors’ specific traits and personal qualities might cause problems. For example, a donor who did not like Aboriginal people or a vegetarian donor organ transplanted to a non-vegetarian. In fact, with the exception of living donors, neither patients nor families usually knew the origin of deceased donor organs, despite their noted interest and speculation. Transplant recipients reported being asked by others about their organ donor, but most reported answering that they did not know.

However, patient and family reservations about transplant were not limited to contested “traditional” values or perceived cultural proscriptions. Families also feared that their family member would be far away at the transplant unit going through a major operation with little support; they worried about their relative surviving. Some patients also expressed similar fears about their own health:

*When I think about my health, I’m diabetic – I don’t know what might happen… I’ve got many things to worry about especially with my health* (2-115).

## Discussion

An overwhelming majority of a large and diverse cohort of Indigenous Australian dialysis patients explicitly expressed a positive – even intense – interest in a transplant. Patients saw it as the only pathway to re-establishing a ‘normal’ life in their homeland with their family; a prospect eclipsing all other potential concerns. However, half of the respondents also described themselves as uninformed about their transplant status and prospects. Most had only a rudimentary knowledge of transplantation with virtually no understanding of eligibility criteria, the pathway to being listed and the risks/benefits associated with transplantation. One in four patients were either mistaken or uncertain about their current listing status.

Factors contributing to patients’ difficulties include multiple linguistic, social and cultural communication barriers, and perceptions of systematic exclusion from critical knowledge in combination with a lack of culturally appropriate, user-friendly information and patient education strategies.

The remoteness and distances characteristic of northern and central Australia means that the majority of Indigenous dialysis patients are hundreds – if not thousands – of kilometres from a transplant unit. Rather than perceiving a pathway of recognisable ‘steps’ towards transplant, patients portray a scenario best characterised as ‘out-of-sight, out-of-mind’. This continues post-transplant as patients typically return to their families in remote regions where threats to health are many but services are few. The poorer transplant outcomes of Indigenous patients, particularly from remote areas, represents an on-going concern for treating nephrologists [15].

Important cultural considerations for Indigenous patients and families contemplating transplant included the integrity of the body, autonomy in relation to one’s body/actions, possible dangers associated with transgressing psychic boundaries, the ethics of directly requesting kin to consider donation, perceived responsibilities of organ recipients for donor and family health, and potential obligation associated with organ donations. No broad consensus was apparent among Indigenous patients as to the relative importance of these many, specific considerations. Individual patients and family members hold a wide variety of views and potentially experience significantly stressful decision-making.

In explaining their intense interest in transplant, Indigenous patients repeatedly referenced the primacy of family, kin and homeland as true sources of well-being and health. Our study also suggests that patients’ interest in transplant may not necessarily align with broader Indigenous community views.

### Study limitations

This study reports on the first comprehensive, systematic exploration of Australian Indigenous (ESKD) patients’ views on transplant. A large number of Indigenous respondents (146) representing diverse social, cultural and treatment contexts gives the findings reliability and generalisability for Australia. However it is likely that the Indigenous patients interviewed represent the more confident and articulate sector of that patient group. Other patients, less confident, perhaps angry, distressed or confused, are unlikely to have volunteered to speak with a stranger. The study therefore almost certainly understates the difficulties Indigenous patients experience in relation to transplant. Access to transplant for Indigenous Australians has barely changed in a decade [4, 5], and this account of Indigenous patients’ views on transplant – *the only such report* – remains highly pertinent.

## Conclusions

Indigenous ESKD patients demonstrated an intense interest in transplantation with a preference for deceased donor organs over living kidney donation. Patients believe transplant is the path most likely to support the re-establishment of a ‘normal’ family life. Patients described themselves as poorly informed with most having a rudimentary understanding of the notion of transplant but no understanding of eligibility criteria, the transplant procedure and associated risks. Patients experienced multiple communication barriers that - taken together - undermine their engagement in treatment decision-making. Their families and communities are disempowered because they also lack information to reach a shared understanding of transplantation. Although cultural sensitivities associated with transplantation were described, these did not appear to constrain patients in making choices about their own health.

### Implications for research and practice

Transplant units and local treatment providers should collaborate to develop user-friendly, culturally informed and region-specific patient education programs and practices for Indigenous ESKD/transplant patients. Using quality improvement cycles, including regular patient feedback, these programs should underpin the development of national guidelines for patient education.

There is also a need to sensitively create or sponsor opportunities for informed transplant-related dialogue **with and within** Indigenous communities to canvass the full range of issues regarding organ donation and transplantation.

An ongoing concern of nephrologists relates to poorer transplant outcomes for Indigenous ESKD patients. This suggests that we need to have a better understanding of factors that predict outcomes. Research to explore patient, service, health system and broader social determinants of outcomes should be prioritised.

It is clear from our study that Indigenous ESKD patients are desperate to reclaim a normal life and to go home. Patients see transplant as a pathway to achieve these aims. In the presence of such extreme inequity in access, we need to ask whether a utilitarian approach to organ allocation guidelines is appropriate. Rather than comparing Indigenous and non-Indigenous transplant outcomes, it would appear fairer to compare the risks and benefits of transplant versus remaining on dialysis for Indigenous patients.

## Additional file

Additional file 1: Human Research Ethics Committees (HREC) approving IMPAKT research described in this report. Description of data: List of HRECs approving IMPAKT research. (DOCX 18 kb)
