# Supplementary material for: Difficult conversations: Australian Indigenous patients’ views on kidney transplantation
Source: BMC Nephrol. 2017 Oct 11;18:310. doi: 10.1186/s12882-017-0726-z (PMC5637064; doi:10.1186/s12882-017-0726-z)
Supplement: Additional file 1: — Human Research Ethics Committees (HREC) approving IMPAKT research described in this report. Description of data: List of HRECs approving IMPAKT research. (DOCX 18 kb) [file 12882_2017_726_MOESM1_ESM.docx]

| **Additional File 1: Human Research Ethics Committees (HREC) approving research described in this report** | |
| --- | --- |
| **CURRENT HREC NAME** | **OTHER IDENTIFICATION DETAILS** |
| Aboriginal Health Research Ethics Committee, South Australia | NHMRC****** Registration EC00185 |
| Department for Health & Ageing Human Research Ethics Committee, South Australia | NHMRC Registration EC00304 |
| Human Research Ethics Committee (TQEH) The Queen Elizabeth Hospital, South Australia | NHMRC Registration EC 00190 |
| Western Australian Aboriginal Health Ethics Committee, Western Australia | NHMRC Registration EC00292 |
| Royal Perth Hospital Ethics Committee, Perth, Western Australia | NHMRC Registration EC00270 |
| Wuchopperen Health Service Ethics Committee, Cairns Queensland | Aboriginal Community Controlled Health Service |
| Far North Queensland Human Research Ethics Committee, Cairns, Queensland (covers Cairns Base Hospital) | NHMRC Registration EC00157 |
| Metro South, Human Research Ethics Committee, Brisbane Queensland (covers Princess Alexandra Hospital) | NHMRC Registration EC 00167 |
| Townsville Hospital and Health Services Human Research Ethics Committee | NHMRC Registration EC00183 |
| Human Research Ethics Committee for Northern Territory Department of Health & Menzies School of Health Research (Darwin) | NHMRC Registration EC 00153 |
| Central Australian Health Research Ethics Committee, Alice Springs, Northern Territory | NHMRC Registration EC00155 |
| Sydney Local Health District (covers Royal Prince Alfred Hospital), New South Wales | NHMRC Registration EC 00113 |
| Greater Western Research Ethics Committee, Western New South Wales Local Health District (covers Dubbo Hospital) | NHMRC Registration EC00399 |
| Aboriginal Health and Medical Research Committee, New South Wales | NHMRC Registration EC00342 |

******National Health and Medical Research Council of Australia (NHMRC): registration indicates that the HREC complies with the NHMRC *National Statement on Ethical Conduct in Human Research 2007*

Source for ID: <https://www.nhmrc.gov.au/_files_nhmrc/file/health_ethics/hrecs/att_2_-_list_of_hrecs_registered_with_nhmrc_february_2017.pdf> Accessed 28 February 2017
